# A Label-Free Aptasensor for Ochratoxin a Detection Based on the Structure Switch of Aptamer

**DOI:** 10.3390/s18061769

**Published:** 2018-06-01

**Authors:** Feng Liu, Ailing Ding, Jiushang Zheng, Jiucun Chen, Bin Wang

**Affiliations:** Institute for Clean Energy and Advanced Materials, Faculty of Materials and Energy, Southwest University, Chongqing 400715, China; swuliufeng@swu.edu.cn (F.L.); dal208@email.swu.edu.cn (A.D.); zjs1993425@email.swu.edu.cn (J.Z.)

**Keywords:** aptamer, OTA, structure switching, ZnPPIX, fluorescence

## Abstract

A label-free sensing platform is developed based on switching the structure of aptamer for highly sensitive and selective fluorescence detection of ochratoxin A (OTA). OTA induces the structure of aptamer, transforms into G-quadruplex and produces strong fluorescence in the presence of zinc(II)-protoporphyrin IX probe due to the specific bind to G-quadruplex. The simple method exhibits high sensitivity towards OTA with a detection limit of 0.03 nM and excellent selectivity over other mycotoxins. In addition, the successful detection of OTA in real samples represents a promising application in food safety.

## 1. Introduction

**Figure 1:**
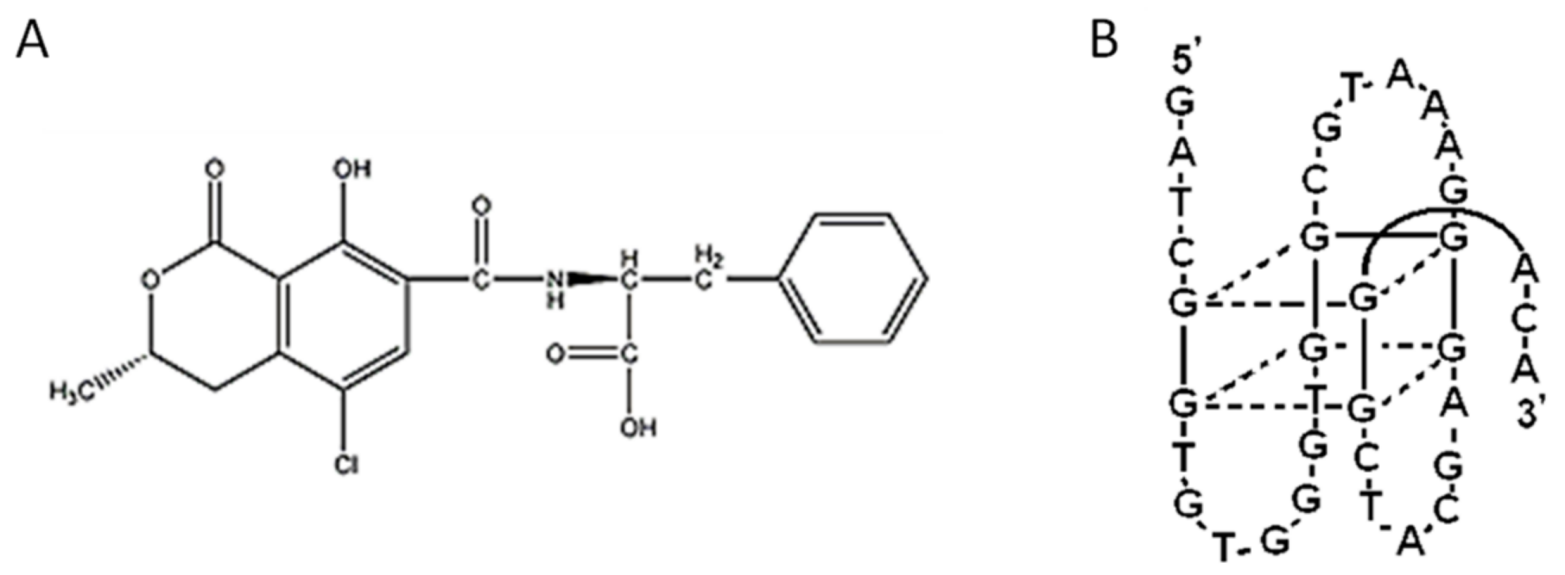
The chemical structure of OTA (**A**) and its aptamer (**B**).

Ochratoxin A (OTA), a mycotoxin generated by different fungi species, such as *Aspergillus* and *Penicillium* (the chemical structure of OTA is shown in Figure 1A), is reported to be a nephrotoxic toxin with strong teratogenic, neurotoxic, immunotoxic, and carcinogenic effects on humans [1,2,3]. OTA contaminates a great variety of agricultural products such as cereals, wheat, corn, beans, coca and wine, threatening people’s lives and causing severe economic losses [4,5]. Therefore, it is necessary to develop a rapid and sensitive analytic technique for OTA detection. 

Common methods used for the detection of OTA include mass spectroscopy, high performance chromatography, and antibody-based techniques [6,7,8]. These methods display excellent stability, accuracy and reliability for the measurement of OTA, but they are time-consuming, complex and require large amounts of equipment and trained operators [9]. Hence, it is vital to develop a rapid, sensitive and easy-to-operate method for OTA detection.

Recently various aptamer-based biosensors are rapidly developed due to the noticeable merits of aptamer over antibody, such as convenient synthesis and modification, high specificity and stability [10]. To improve the sensitivity of the sensor, a variety of nanomaterials (e.g., AuNPs, magnetic nanoparticles, silica nanoparticles, graphene, quantum dots, carbon dots, etc. [10,11,12,13,14]) and signal amplified techniques (e.g., loop-mediated isothermal amplification (LAMP), hybridization chain reaction (HCR), rolling-circle amplification (RCA) etc. [15,16,17]) are utilized to improve the sensitivity of OTA detection. These methods and techniques greatly improve the detection performance of the sensor towards OTA but the requisite label and treatment process increases their operation difficulty and cost, which seriously affects their potential application.

Aptamers are DNA or RNA oligonucleotides which can be synthesized by a simple chemical method. Like antibodies, aptamers can specifically bind to a variety of targets with high selectivity [18]. Various OTA sensors are developed for OTA detection, which confirms the feasibility of the method in practical applications [19,20,21]. It was reported that the aptamer of OTA transduces into an antiparallel G-quadruplex structure in the presence of OTA (as shown in Figure 1B) [13,22,23]. Based on the theory, different aptasensors, including luminescent [24,25,26], colorimetric [27], and electrochemical [28,29] methods, are developed for OTA detection. However, most of these methods require oligonucleotides labelling and nanomaterials modification, which result in complex procedures, time dependence and application limitation. For example, Yu et al. [30] developed an exonuclease-assisted multicolor aptasensor for the visual detection of OTA. In their design, a DNA sequence comprising an OTA aptamer and a hemin aptamer was used for OTA recognition and signal conversion. OTA was quantified based on Exonuclease I (Exo I)-based amplification and DNA enzyme-hemin, which catalyzed the oxidation of 3,3′,5,5′-tetramethylbenzidine (TMB). Using this method, OTA can be detected by visual observation. However, the experimental procedure is too complex and largely restricts its wide application. Hence the development of simple, rapid and low- cost detection technology is indispensable. Lu and his coworker [31] developed a simple method for OTA detection based on switching the structure of aptamer. In their design, partially hybridized OTA aptamer was transformed into a G-quadruplex structure in the presence of OTA, and OTA was quantified by the G-quadruplex specific of Ir(III) complex probe. The method is advantageous due to the simple procedure, low cost and notable selectivity. However, its sensitivity is low (its detection limit is 5 nM towards OTA). Therefore, the development of a suitable OTA sensor is still a tremendous challenge.

A new structure-switching sensing platform was developed based on OTA aptamer and zinc(II)-protoporphyrin IX (ZnPPIX) fluorophore. We found that the sensitivity of the aptasensor could be increased about 100 times by replacing Ir(III) complex with ZnPPIX probe for OTA detection. 

## 2. Experimental Section

### 2.1. Materials and Apparatus

Oligonucleotides were chemically synthesized by Shanghai Sangon Biotechnology Co., Ltd. (Shanghai, China) and purified by PAGE. The sequences of the oligonucleotides are as follows: apt-OTA (OTA aptamer): GAT CGG GTG TGG GTG GCG TAA AGG GAG CAT CGG ACA [32]; a randomly selected control oligonucleotide (cDNA): TCA GTG CCT AGC GAA GTG CAC GAA TCG AGA CGT AGT. Protoporphyrin IX Zinc(II) (ZnPPIX), Aflatoxin A1 (AFB1) and ochratoxin A (OTA) were purchased from Sigma-Aldrich and used without further purification. Ochratoxin B (OTB), Zearalenone, and Fumidil were purchased from Leon Technology Co. Ltd. (Beijing, China). Stock solutions of all mycotoxins were prepared by dissolving them in 60% methanol. All other chemicals were of analytical grade. Deionized water was purified with a Milli-Q Synthesis A10 water purification system and used throughout the experiments. Fluorescence measurements were performed on a Shimadzu RF-5301PC spectro-fluorometer equipped with a 150 W xenon lamp as the excitation source (Ushio Inc., Tokyo, Japan).

### 2.2. Fluorescence Measurements of OTA

Before measurements, the stock solutions of the aptamer were incubated at 82 °C for 10 min and natural cooled to room temperature. For OTA measurement, 10 µL of 2 µM aptamer stock solution was mixed with 10 µL of 20 nM OTA methanol solution. After incubation for 10 min, the mixture was added into 170 µL of 50 mM HEPES buffer solution (pH 7.0, containing 50 mM NaCl). Then 10 µL of ZnPPIX solution was added and incubated at room temperature for 10 min. The emission spectra of these samples were recorded at am excitation wavelength of 430 nm. This was repeated at least three times.

### 2.3. Optimization of OTA Measurements

To obtain the best detection results, the concentration of aptamer and ZnPPIX were optimized. Firstly, the concentration of aptamer was optimized following the same procedure above. Different concentration of aptamer stock solution was mixed with 10 µL of 20 nM OTA methanol solution. After incubating for 10 min, the mixture was added into 170 µL of 50 mM HEPES buffer solution (pH 7.0, containing 50 mM NaCl). Then, 10 µL of ZnPPIX solution was added and incubated at room temperature for 10 min, and the emission spectra were recorded at excitation wavelength of 430 nm. Following the same procedure above, the concentration of ZnPPIX was explored using 160 nM aptamer and different concentration of ZnPPIX from 0.08 to 0.96 µM. 

### 2.4. Selectivity of OTA Measurements

The selectivity against common toxins was carried out to explore the anti-interference performance of the method. Ten microliters of aptamer stock solution were mixed with the same concentration of OTA, OTB, AFB1, fumidil and zearalenone solution respectively. After incubating for 10 min, the mixture was added to 170 µL of 50 mM HEPES buffer solution (pH 7.0, containing 50 mM NaCl). Then, 10 µL of ZnPPIX solution was added and incubated at room temperature for 10 min, followed by fluorescence determination at excitation wavelength of 430 nm.

### 2.5. Detection of OTA in Real Food Samples

Corn flour samples were purchased from the local supermarket. Aliquots of the corn flour were mixed with OTA at different concentrations and extracted with 60% (*v*/*v*) methanol. After centrifugation at 10,000 rpm for 10 min, the supernatant was taken and determined by the proposed method. Typically, 10 μL of 1 μM aptamer was mixed with 20 μL of 100 mM HEPES (pH 7.0, 100 mM NaCl) buffer solution and 10 μL of the prepared corn samples. After incubating at room temperature for 10 min, 13 μL of ZnPPIX solution (10 µM) was added and the final volume was adjusted to 200 μL with water. Then, fluorescent spectra of the samples were recorded at an excitation wavelength of 430 nM.

## 3. Results and Discussion

### 3.1. Scheme of OTA Detection

**Scheme 1:**
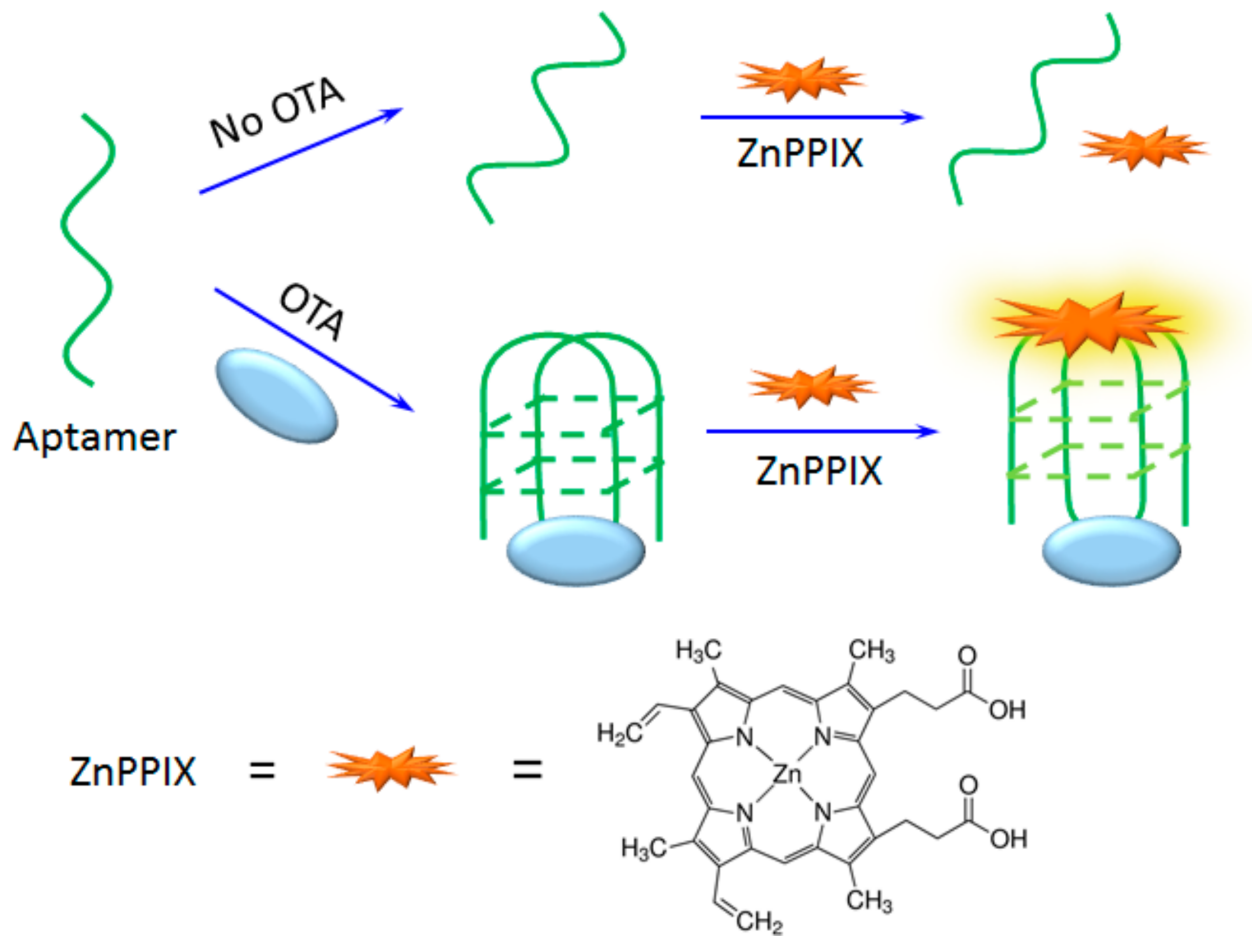
Scheme of the label free OTA detection based on structure switching of aptamer.

It was reported that OTA aptamer transforms into G-quadruplex structure in the presence of OTA, which can be used for the detection of OTA under the assistance of G-quadruplex specific fluorophore [31]. For fluorescent analysis, the common G-quadruplex ligands include Thioflavin T, *N*-methyl-mesoporphyrin (NMM) and ZnPPIX [33,34], however only ZnPPIX displayed sensitive response signals in the presence of OTA. Hence, ZnPPIX is employed here for the detection of OTA. Scheme 1 shows the design of OTA detection based on the structure switch of OTA aptamer using a ZnPPIX probe. In an aqueous solution, ZnPPIX does not bind with aptamer or display obvious fluorescence. In the presence of OTA, the aptamer binds specifically with OTA and transforms the structure of aptamer into a G-quadruplex structure. Then, the ZnPPIX probe efficiently binds with G-quadruplex and produces strong fluorescence, and OTA can be quantified based on the enhanced fluorescence. 

**Figure 2:**
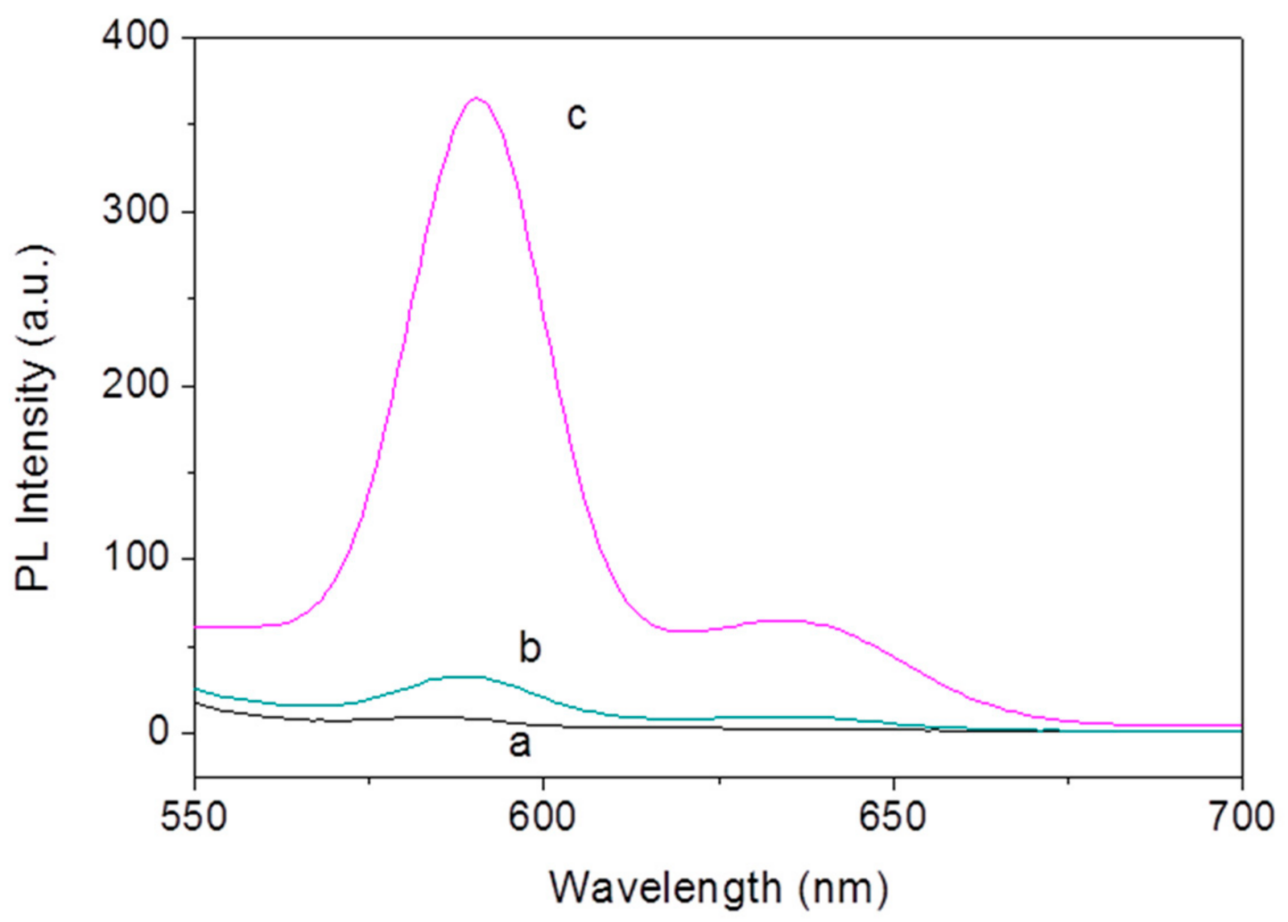
The emission spectra of ZnPPIX (**a**), ZnPPIX + aptamer (**b**), and ZnPPIX + aptamer + OTA (**c**).

To verify the design, we researched the fluorescence property of the OTA-aptamer-ZnPPIX sensing system. As shown in Figure 2, ZnPPIX shows negligible fluorescence in pH 7.0 HEPES buffer solution (curve a). Upon adding the aptamer the fluorescence of the mixture only slightly increased (curve b). However, if the aptamer is mixed with OTA and incubated for 10 min before the addition of ZnPPIX, the fluorescence of the sensing system is greatly enhanced (curve c in Figure 2). The results demonstrate the feasibility of our design. 

### 3.2. Optimization of OTA Detection

**Figure 3:**
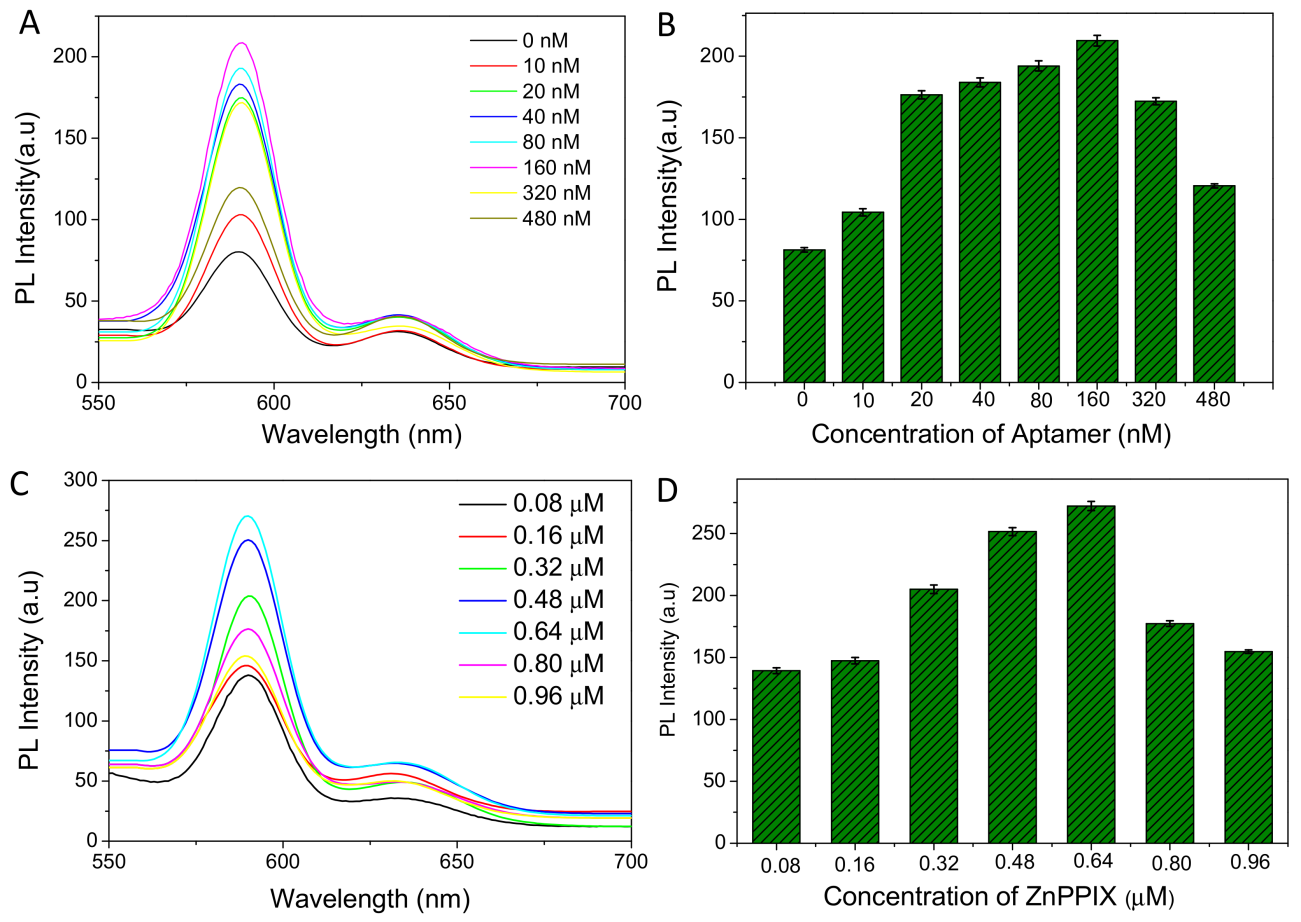
(**A**) The emission spectra of aptamer-ZnPPIX system containing 0.64 µM ZnPPIX, 1 nM OTA and different concentration of aptamer; (**B**) The relationship of fluorescence response and aptamer concentration; (**C**) The emission spectra of aptamer-ZnPPIX system containing 160 nM aptamer, 1 nM OTA and different concentration of ZnPPIX probe; (**D**) The relationship of fluorescence response and ZnPPIX concentration. (λex = 430 nm, 10 mM HEPES (pH 7.0, containing 50 mM NaCl).

To obtain the best detection result, the effect of ZnPPIX and aptamer on OTA determination is studied. Firstly, the concentration of aptamer was optimized. The structure transformation from aptamer to G-quadruplex is a chemical equilibrium, so the concentration of aptamer greatly affects the formation of G-quadruplex in the presence of OTA. If the concentration of aptamer is too low, a higher concentration of OTA is needed to facilitate the formation of G-quadruplex, which results in decreased sensitivity of OTA determination. Conversely, an excessively-high concentration of aptamer would lead to high background signal. As shown in Figure 3A,B, when 1 nM OTA was mixed with different concentrations of aptamer, the addition of ZnPPIX caused a different fluorescent response in 10 mM HEPES buffer solution (pH 7.0). When the concentration of aptamer increased from 0 to 160 nM, the fluorescence of ZnPPIX enhances gradually and the strongest fluorescence is obtained upon the addition of 160 nM ZnPPIX. Next, the effect of ZnPPIX concentration on the sensing of OTA was explored. As shown in Figure 3C,D, in 10 mM HEPES buffer solution (pH 7.0, containing 10 mM NaCl, 1 nM OTA and 160 nM aptamer), the fluorescent intensity of the system increases dramatically with the addition of ZnPPIX in the concentration range from 0.08 to 0.64 µM. The fluorescence intensity reached its the maximal value when the concentration of ZnPPIX is increased to 0.64 µM, which was used as the optimized concentration for the OTA detection. All OTA determinations are carried out under the optimized conditions.

### 3.3. Quantitative Measurement of OTA

**Figure 4:**
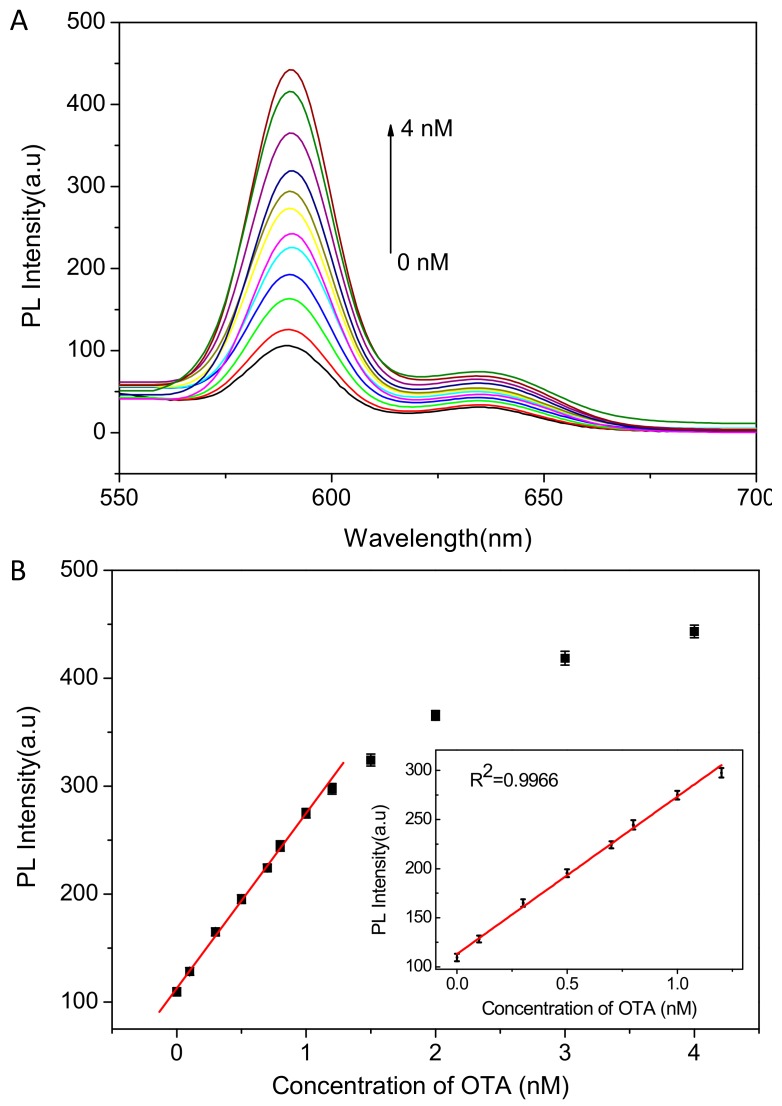
(**A**) Emission spectra of the assay system with different concentrations of OTA from 0~4 Nm; (**B**) The relationship between fluorescent intensity and OTA concentration. Inset: linear plot of fluorescent intensity vs. OTA concentration. Error bars show the standard deviations of three independent measurements (λex = 430 nm).

Under the optimized condition, OTA was determined by the proposed method. As shown in Figure 4, with the increase of OTA concentration, the fluorescence of ZnPPIX increased gradually in 10 mM HEPES solution (pH 7.0, 10 mM NaCl). A linear relationship between the fluorescence intensity and the concentration of OTA is obtained in the range of 0.1~1.2 nM. The small linear range can be attributed to the higher concentration of ZnPPIX probe which results in part fluorescence quenching by self-absorption effect. The linear regression equation is *I* = 158.36*c* + 113.89 (R^2^ = 0.9966), where *I* is the fluorescence intensity and *c* is the concentration of OTA (nM). The limit of detection is calculated to be about 0.03 nM based on the signal to noise ratio of 3 (S/N = 3). The results demonstrate that the detection platform displayed higher sensitivity than most of the previous reports (as shown in Table 1). Owing to the higher sensitivity, the real samples containing higher concentration of OTA can also be detected by dilution. Hence, the method can be applied for OTA detection in most real samples. In addition, the proposed method is simple, label-free and cost-effective, which facilitates its wide application in biosensing and food safety. These results reveal that the developed aptasensor has great potential application in the sensitive detection of OTA in practical samples.

### 3.4. Selectivity of Structure-Switching Aptasensor

**Figure 5:**
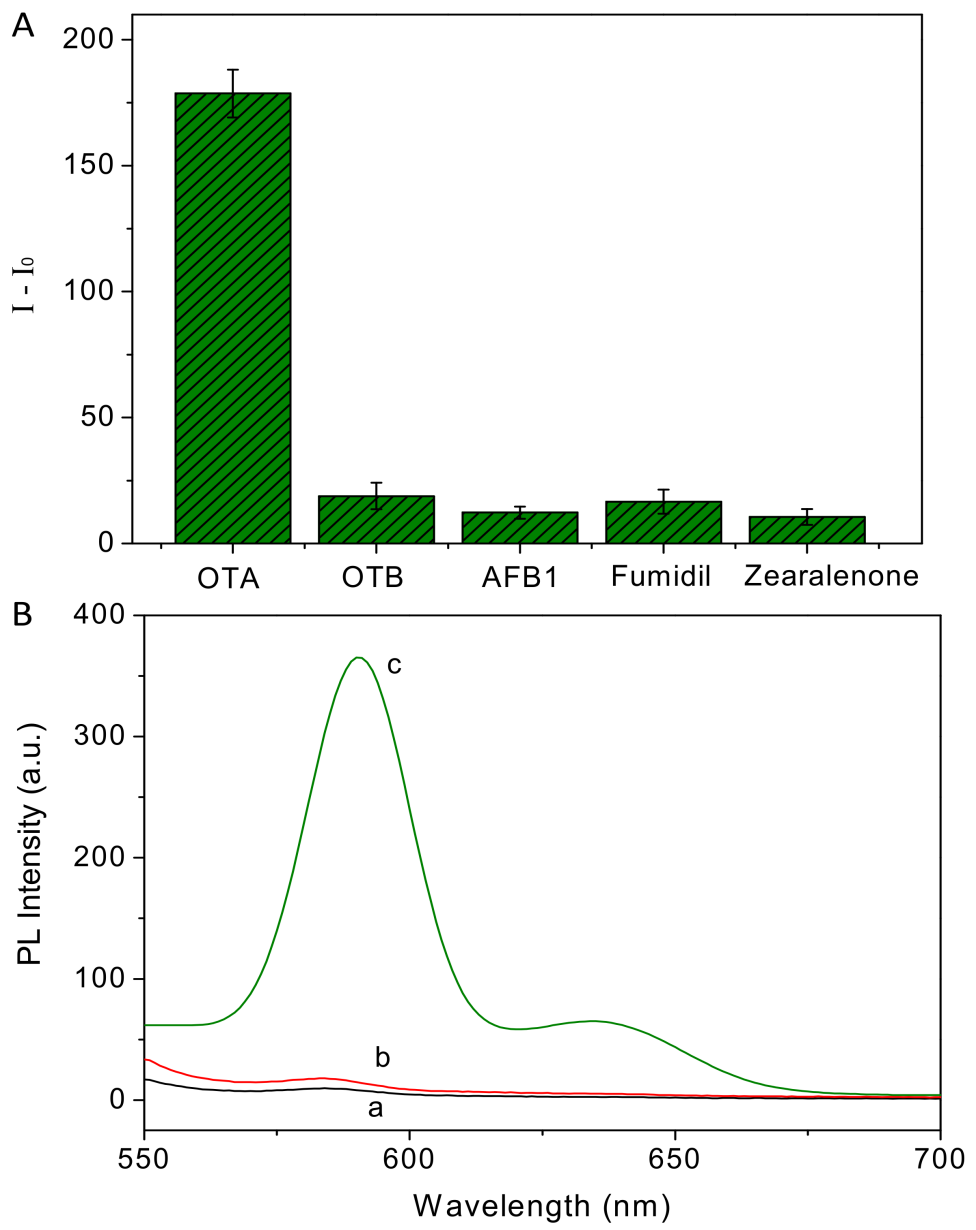
(**A**) Selectivity of the detection method to OTA over its analogues (the concentration of all mycotoxins are 1 nM); (**B**) emission spectra of ZnPPIX (a), ZnPPIX + cDNA + OTA (b), and ZnPPIX + aptamer + OTA (c).

In order to evaluate the selectivity of the structure-switching aptasensor, we tested the sensing platform against various common analogues, including ochratoxin B (OTB), aflatoxin B1 (AFB1), Fumidil, and Zearalenone. As shown in Figure 5A, the addition of target molecule (OTA) causes dramatic fluorescence enhancement. However, other mycotoxins at the same concentration do not induce apparent fluorescent intensity increase, indicating the high specificity to OTA. In order to explore the high selectivity of the sensing platform, a control DNA (cDNA) with random sequence is employed to replace aptamer for OTA determination. From the results in Figure 5B we can see that the cDNA-OTA system cannot cause a significant fluorescence response, confirming that the high selectivity can be ascribed to the specific recognition of aptamer towards OTA.

### 3.5. Determination of OTA in Corn and Red Wine Samples

To evaluate the feasibility of the sensing system for practical applications, OTA determination was performed by standard addition methods in real corn and red wine samples. After corn samples spiked with OTA are extracted with 60% (*v*/*v*) methanol aqueous solution and centrifuged for 10 min, the obtained supernatant solutions are mixed with aptamer and incubated at room temperature for 10 min. Then 0.64 μM ZnPPIX was added for fluorescence determination. The results in Table 2 indicate that the recoveries of the determination are between 96.6% and 106% and the relative standard deviation (RSD) is lower than ±6.1%. The detection in red wine samples also reveal that the recoveries are higher than 94% and the RSD is lower than ±7.3%, suggesting the reliability and feasibility of the method for the detection of OTA in real samples. In addition, from the results in Table 2 we can see that there is a certain difference in OTA detection in different samples, which may result from the complex composition of practical samples.

## 4. Conclusions

In summary, a simple and sensitive aptamer-based sensing platform was developed for the detection of OTA based on switching the structure of aptamer. The sensing system consisted of G-quadruplex probe (ZnPPIX) and OTA aptamer that specifically recognizes and quantifies OTA. The aptamer sensing platform displays higher sensitivity than most reported methods and robust selectivity over other mycotoxins. The successful detection of OTA in real samples confirmed the potential application in food safety. This proposed aptasensor is simple, quick and cost-effective compared to conventional chromatography and immunoassay, which is advantageous to its development and wide application.

## Figures and Tables

**Table 1:** Comparison of the properties of various OTA aptasensor.

Methods	Detection Technology	Detection Limit (nM)	Application	Reference
Electrochemical	Polyaniline Langmuir–Blodgett film based aptasensor	0.25	--	[35]
Electrochemical	Impedimetric Aptasensor	0.014	wine	[36]
Colorimetric	ABTS-H_2_O_2_-Hemin colorimetric system	1	--	[37]
Colorimetric	DNA amplification (Hybridization chain reaction)	0.01	wheat flour	[27]
Fluorescence	Aptamer structure switch with Iridium(III) Complex	0.05	--	[31]
Fluorescence	Fluorophore labeled aptamer structure switching	1.99	corn	[23]
Fluorescence	Aptamer structure switch with ZnPPIX probe	0.03	corn	This work

**Table 2:** Determination of OTA in corn and red wine samples with the proposed method.

Samples		Added OTA (nM)	Found OTA (nM) ^a^	Recovery (%) ^a^	RSD (%) ^a,b^
corn	1	0	0	--	±3.2
	2	0.2	0.21	106	±5.8
	3	0.5	0.48	96	±3.5
	4	1	0.97	97	±6.1
Red wine	1	0	0.05	--	±2.3
	2	0.2	0.19	96	±4.6
	3	0.5	0.47	94	±7.3
	4	1	0.95	95	±6.2

^a^ The mean of three measurements. ^b^ RSD = The relative standard deviation.

## References

[1] Bostan H.B., Danesh N.M., Karimi G., Ramezani M., Shaegh S.A.M., Youssefif K., Charbgoo F., Abnous K., Taghdisi S.M. (2017). Ultrasensitive detection of ochratoxin A using aptasensors. Biosens. Bioelectron..

[2] O’Brien E., Dietrich D.R. (2005). Ochratoxin A: The continuing enigma. Crit. Rev. Toxicol..

[3] El Khoury A., Atoui A. (2010). Ochratoxin A: General Overview and Actual Molecular Status. Toxins.

[4] Chrouda A., Sbartai A., Baraket A., Renaud L., Maaref A., Jaffrezic-Renault N. (2015). An aptasensor for ochratoxin A based on grafting of polyethylene glycol on a boron-doped diamond microcell. Anal. Biochem..

[5] Pfohl-Leszkowicz A., Mandervill R.A. (2007). Ochratoxin A: An overview on toxicity and carcinogenicity in animals and humans. Mol. Nutr. Food Res..

[6] Roland A., Bros P., Bouisseau A., Cavelier F., Schneider R. (2014). Analysis of ochratoxin A in grapes, musts and wines by LC-MS/MS: First comparison of stable isotope dilution assay and diastereomeric dilution assay methods. Anal. Chim. Acta.

[7] Mariño-Repizo L., Kero F., Vandell V., Senior A., Sanz-Ferramola M.I., Cerutti S., Raba J. (2015). A novel solid phase extraction—Ultra high performance liquid chromatography–tandem mass spectrometry method for the quantification of ochratoxin A in red wines. Food Chem..

[8] Pussemier L., Piérard J.-Y., Anselme M., Tangni E., Motte J.-C., Larondelle Y. (2006). Development and application of analytical methods for the determination of mycotoxins in organic and conventional wheat. Food Addit. Contam..

[9] Viter R., Savchuk M., Iatsunskyi I., Pietralik Z., Starodub N., Shpyrka N., Ramanaviciene A., Ramanavicius A. (2018). Analytical, thermodynamical and kinetic characteristics of photoluminescence immunosensor for the determination of Ochratoxin A. Biosens. Bioelectron..

[10] Kim Y.S., Raston N.H.A., Gu M.B. (2016). Aptamer-based nanobiosensors. Biosens. Bioelectron..

[11] Jiang L., Qian J., Yang X., Yan Y., Liu Q., Wang K., Wang K. (2014). Amplified impedimetric aptasensor based on gold nanoparticles covalently bound graphene sheet for the picomolar detection of ochratoxin A. Anal. Chim. Acta.

[12] Taghdisi S.M., Danesh N.M., Beheshti H.R., Ramezanib M., Abnous K. (2016). A novel fluorescent aptasensor based on gold and silica nanoparticles for the ultrasensitive detection of ochratoxin A. Nanoscale.

[13] Zhang J., Zhang X., Yang G., Chen J., Wang S. (2013). A signal-on fluorescent aptasensor based on Tb3þ and structure-switching aptamer for label-free detection of Ochratoxin A in wheat. Biosens. Bioelectron..

[14] Lu Z., Chen X., Hu W. (2017). A fluorescence aptasensor based on semiconductor quantum dots and MoS2 nanosheets for ochratoxin A detection. Sens. Actuators B Chem..

[15] Wang S., Zhang Y., Pang G., Zhang Y., Guo S. (2017). Tuning the Aggregation/Disaggregation Behavior of Graphene Quantum Dots by Structure-Switching Aptamer for High-Sensitivity Fluorescent Ochratoxin A Sensor. Anal. Chem..

[16] Xie S., Chai Y., Yuan Y., Bai L., Yuan R. (2014). Development of an electrochemical method for Ochratoxin A detection based on aptamer and loop-mediated isothermal amplification. Biosens. Bioelectron..

[17] Wang B., Wu Y., Chen Y., Weng B., Xu L., Li C. (2016). A highly sensitive aptasensor for OTA detection based on hybridization chain reaction and fluorescent perylene probe. Biosens. Bioelectron..

[18] Wang B., Yu C. (2010). Fluorescence Turn-On Detection of a Protein through the Reduced Aggregation of a Perylene Probe. Angew. Chem. Int. Ed..

[19] Yang C., Wang Y., Marty J.L., Yang X. (2011). Aptamer-based colorimetric biosensing of Ochratoxin A using unmodified gold nanoparticles indicator. Biosens. Bioelectron..

[20] Mishra R.K., Hayat A., Catanante G., Ocana C., Marty J.L. (2015). A label free aptasensor for Ochratoxin A detection in cocoa beans: An application to chocolate industries. Anal. Chim. Acta.

[21] Hayat A., Mishra R.K., Catanante1 G., Marty J.L. (2015). Development of an aptasensor based on a fluorescent particles-modified aptamer for ochratoxin A detection. Anal. Bioanal. Chem..

[22] Huang L., Wu J.J., Zheng L., Qian H.S., Xue F., Wu Y.C., Pan D.D., Adeloju S.B., Chen W. (2013). Rolling Chain Amplification Based Signal-Enhanced Electrochemical Aptasensor for Ultrasensitive Detection of Ochratoxin A. Anal. Chem..

[23] Chen J., Fang Z., Liu J., Zeng L. (2012). A simple and rapid biosensor for ochratoxin A based on a structure-switching signaling aptamer. Food Control.

[24] Sheng L., Ren J., Miao Y., Wang J., Wang E. (2011). PVP-coated graphene oxide for selective determination of ochratoxin A via quenching fluorescence of free aptamer. Biosens. Bioelectron..

[25] Sharma A., Hayat A., Mishra R.K., Catanante G., Shahid S.A., Bhand S., Marty J.L. (2016). Design of a fluorescence aptaswitch based on the aptamer modulated nano-surface impact on the fluorescence particles. RSC Adv..

[26] McKeague M., Velu R., Hill K., Bardoczy V., Meszaros T., DeRosa M.C. (2014). Selection and Characterization of a Novel DNA Aptamer for Label-Free Fluorescence Biosensing of Ochratoxin A. Toxins.

[27] Wang C., Dong X., Liu Q., Wang K. (2015). Label-free colorimetric aptasensor for sensitive detection of ochratoxin A utilizing hybridization chain reaction. Anal. Chim. Acta.

[28] Rhouati A., Hayat A., Hernandez D.B., Meraihi Z., Munoz R., Marty J.L. (2013). Development of an automated flow-based electrochemical aptasensor for on-line detection of ochratoxin A. Sens. Actuators B Chem..

[29] Chen Y., Yang M.L., Xiang Y., Yuan R., Chai Y.Q. (2014). Binding-induced autonomous disassembly of aptamer–DNAzyme supersandwich nanostructures for sensitive electrochemiluminescence turn-on detection of ochratoxin A. Nanoscale.

[30] Yu X., Lin Y., Wang X., Xu L., Wang Z.W., Fu F.F. (2018). Exonuclease-assisted multicolor aptasensor for visual detection of ochratoxin A based on G-quadruplex-hemin DNAzyme-mediated etching of gold nanorod. Microchim. Acta.

[31] Lu L., Wang M., Liu L., Leung C., Ma D.L. (2015). Label-Free Luminescent Switch-On Probe for Ochratoxin A Detection Using a G-Quadruplex-Selective Iridium(III) Complex. ACS Appl. Mater. Interfaces.

[32] Cruz-Aguado J.A., Penner G. (2008). Determination of Ochratoxin A with a DNA Aptamer. J. Agric. Food Chem..

[33] Zhang Z., Sharon E., Freeman R., Liu X., Willner I. (2012). Fluorescence Detection of DNA, Adenosine-5′-Triphosphate (ATP), and Telomerase Activity by Zinc(II)-Protoporphyrin IX/G-Quadruplex Labels. Anal. Chem..

[34] Yu Z., Zhou W., Han J., Li Y., Fan L., Li X. (2016). Na^+^-Induced Conformational Change of Pb^2+^-Stabilized G-Quadruplex and Its Influence on Pb^2+^ Detection. Anal. Chem..

[35] Prabhakar N., Matharu Z., Malhotra B.D. (2011). Polyaniline Langmuir–Blodgett film based aptasensor for ochratoxin A detection. Biosens. Bioelectron..

[36] Rivas L., Mayorga-Martinez C.C., Quesada-González D., Zamora-Gálvez A., de la Escosura-Muñiz A., MerkoçI A. (2015). Label-Free Impedimetric Aptasensor for Ochratoxin-A Detection Using Iridium Oxide Nanoparticles. Anal. Chem..

[37] Lee J., Jeon C.H., Ahnc S.J., Ha T.H. (2014). Highly stable colorimetric aptamer sensors for detection of ochratoxin A through optimizing the sequence with the covalent conjugation of hemin. Analyst.

